# Influence of acidified-biochar on phosphorus and potassium availability in alkaline sandy soil

**DOI:** 10.1038/s41598-025-16247-3

**Published:** 2025-08-20

**Authors:** Tamer A. Elbana, Noura Bakr, Sahar A. Shahin, Nahed A. A. Azab, Soad M. El-Ashry

**Affiliations:** Soils and Water Use Department, Agricultural and Biological Research Institute, National Research Centre, Cairo, Egypt

**Keywords:** Acidification, Soil incubation, Olsen-extractable p, Exchangeable k, Superphosphate, Ecology, Ecology, Environmental sciences

## Abstract

Biochar application is recommended to enhance soil fertility, particularly in arid and semi-arid regions. In alkaline soils, acidifying biochar can help overcome high pH constraints and improve phosphorus (P) and potassium (K) availability. This study evaluated the effects of acidified palm frond (PF) and guava branch (GB) biochars on P and K availability in alkaline sandy soil through a controlled batch incubation experiment. Biochars were acidified using acetic or phosphoric acid and characterized for their chemical properties, surface morphology, and functional groups. A 15-week incubation was conducted using sandy alkaline soil pre-treated with 0.48 Mg ha^-1^ of superphosphate fertilizer. Acidified and unacidified PF and GB biochars were applied at rates of 2.4 and 4.8 Mg ha^-1^. PF biochar showed a higher cation exchange capacity (CEC: 37.9 cmol kg^-1^) and greater K enrichment than GB biochar (CEC: 17.6 cmol kg^-1^). Phosphoric acid significantly increased the CEC of both biochars, while acetic acid had minimal impact. Application of 4.8 Mg ha^-1^ phosphoric acid-treated GB biochar significantly improved soil P availability, reaching 104.02 mg kg^-1^ after one week, with no evidence of P fixation over 15 weeks. PF biochar, regardless of acidification, significantly enhanced exchangeable K levels, particularly at the higher application rate. Acidification improved P and K availability in the studied alkaline sandy soil, but such influence depended on biochar type, chemical composition, and incubation time. Selecting the appropriate acid for biochar modification is crucial to optimizing nutrient availability in alkaline soils.

## Introduction

Incorporating crop residues into soils increases soil organic carbon (SOC) and enhances productivity. Biochar, a carbon-rich material with high resistance to degradation, is recommended for sustainable agriculture as it supports climate change mitigation (SDG 13) and combats land degradation (SDG 15). Biochar addition to soils can provide 22–27% greater climate mitigation than using the same biomass for energy^1^. A nine-year field study showed SOC increases of 23.7% with maize straw and 40.5% with its biochar, which also raised annual SOC accumulation from 0.16 to 0.91 Mg C ha⁻¹ yr⁻¹ and improved yields^2,3^. When combined with effective fertilizer management, biochar further enhances yields by improving fertility, nutrient retention, SOC, and cation exchange capacity (CEC), especially in coarse-textured soils^4,5^.

Phosphorus (P) availability in agricultural soils is often a limiting factor for crop productivity, particularly under alkaline soil conditions. Elbasiouny et al.^6^ attributed the low P availability (Olsen-extractable P ranging from 0.12 mg kg⁻¹ to 18.0 mg kg⁻¹) in the northern Nile Delta of Egypt to soil alkalinity and the strong sorption of P onto calcite minerals. Similarly, Bertrand et al.^7^ found that in alkaline soils of southern Australia cultivated for over 50 years, P sorption was positively correlated with CaCO₃ content and negatively correlated with the concentrations of Fe, Al, and Mn oxides, as well as clay content. Various agricultural practices have been implemented to enhance P availability in alkaline soils, including the application of organic fertilizers^8,9^, P-enriched biochar^10^, and other soil amendments.

Potassium (K) is an essential nutrient that plays a vital role in plant growth and drought tolerance. Various management practices have been developed to enhance K availability and minimize its leaching from soils. For instance, the addition of zeolite and compost has been shown to improve K retention in alkaline sandy soils^11^, while organic fertilizers have proven effective in increasing K availability in alkaline clay soils^12^. In a three-year field study, Wang et al.^13^ reported that combining K-solubilizing bacteria with natural K-feldspar increased K availability by 18% and fruit yield by over 20% in alkaline sandy soils. More recently, biochar application has gained attention for enhancing K availability and plant uptake, with its efficacy influenced by factors such as soil characteristics, feedstock source, biochar aging, and pyrolysis conditions^14^.

Biochar can enhance soil fertility, but its effects on phosphorus (P) and potassium (K) availability vary with feedstock, properties, application rate, and soil conditions^15^. It may increase P availability by modifying soil pH, cation exchange capacity (CEC), and adsorption capacity, thereby enriching active inorganic P forms^16,17^, with stronger effects in acidic and neutral soils than in alkaline soils^18^. Applying alkaline biochar to acidic soils and acidified biochar to alkaline soils is recommended. However, biochar without added P fertilizer can reduce P availability, whereas P-laden biochar often outperforms soluble P fertilizers^10,19^. For K, biochar affects both release and leaching, with finer particles releasing K more rapidly^20^. Its influence on soluble, exchangeable, and non-exchangeable K depends on soil type and crop stage, as shown in wheat–maize rotations where patterns differed between Alfisols and Entisols^21^.

While biochar has been widely studied for improving soil fertility, its effects on nutrient availability in alkaline soils remain poorly understood. This study addresses this gap by asking: Can acid-modified biochar enhance phosphorus (P) and potassium (K) availability in alkaline sandy soils more effectively than unmodified biochar? We hypothesize that acidifying biochars derived from palm fronds and guava branches, abundant feedstocks in arid agroecosystems, will increase P and K availability compared to their unmodified forms.

## Materials and methods

A surface sandy soil (0–20 cm) was collected from the experimental research station of the National Research Centre in Nubaria, Egypt. The soil is classified as Typic Torripsamment^22^. The collected soil was air-dried and was sieved through a 2 mm mesh for use in incubation experiments. Soil pH and electrical conductivity (EC) were measured in a 1:1 soil-to-distilled water suspension after shaking for 1 h and 24 h, respectively^23^. The CaCO₃ equivalent and loss-on-ignition (LOI) were determined according to^24,25^, respectively.

Moreover, two different biochar materials were collected from the local Egyptian agricultural market for use in this study: palm fronds biochar (PF) from El Wadi El Gedid and guava branches biochar (GB) from the Wadi El Natrun region. The biochar materials were ground and passed through 2-mm sieve. Eight acidified biochar materials were prepared using either 0.25 *M* and 0.50 *M* of acetic acid or phosphoric acid. Acidification was carried out by mixing biochar and acid solution in a 1:2 (g: mL) ratio. Therefore, the following treatments were produced: 0.25 acetic-PF, 0.5 acetic-PF, 0.25 phosphoric-PF, 0.5 phosphoric-PF, 0.25 acetic-GB, 0.5 acetic-GB, 0.25 phosphoric-GB, and 0.5 phosphoric-GB. The modified biochar preserved for two weeks until their weight stabilized, prior to further analysis and use.

### Characterization of Biochar materials

#### Chemical analyses

The biochar materials were analyzed for pH in a distilled water suspension (3 g: 30 mL) that was agitated for 1 h using a reciprocal shaker at 200 rpm. Salinity, along with soluble Na, K, and Ca, was determined using a 1:10 biochar-to-water suspension shaken for 24 h. The unmodified biochar materials were digested by concentrated sulfuric and perchloric acids to extract total Na, K, and Ca cations. A Jenway PFP7 flame photometer (Cole-Parmer Ltd, Stone, Staffs, UK) was utilized for measuring total and soluble concentrations of cations. The CEC of biochar materials was measured using ammonium acetate (NH4-OAc, pH 7.0) method^26^.

### Scanning electron microscope (SEM) analyses

Scanning electron microscopy (SEM) was conducted to examine the morphology of both unmodified and acidified biochar using a Thermo Fisher Quanta FEG 250, equipped with a field emission gun (FEG) and operated at 20 kV. Samples were prepared using an S150A Sputter Coater to ensure high-quality micrographs. Additionally, energy-dispersive X-ray spectroscopy (EDX) was performed to determine the elemental composition of the unmodified biochar materials.

#### Fourier-Transform infrared analysis

Attenuated Total Reflectance Fourier-Transform Infrared (ATR-FTIR) spectroscopy was performed using a Bruker VERTEX 80 (Germany) equipped with a Platinum Diamond ATR accessory. The system uses a diamond crystal as the internal reflector and operates within a spectral range of 4000–400 cm⁻¹ at a resolution of 4 cm⁻¹. The crystal has a refractive index of 2.4. FTIR spectral interpretation was conducted based on^27,28^. IrAnalyze software (version 7.1.5.0, labCognition Analytical Software) was employed to identify absorption bands and their corresponding functional groups.

### Incubation experiments

Superphosphate (13% P₂O₅) was applied at a rate of 0.48 Mg ha⁻¹ to the sandy soil for the incubation experiment, reflecting common agricultural practices in sandy soils of Egypt. In triplicate, the experiment conducted in a complete random design to investigate the effects of two application rates (2.4 and 4.8 Mg ha⁻¹) of either unmodified or acidified biochar on P availability. Specifically, unmodified biochars PF and GB along with their acidified counterparts treated with 0.25 *M* acetic or phosphoric acid (0.25 acetic-PF, 0.25 phosphoric-PF, 0.25 acetic-GB, and 0.25 phosphoric-GB) were included in the incubation study. Based on the chemical characterization of the modified biochars, the use of a 0.25 *M* acid concentration was selected for the incubation experiments as an economical and environmentally sustainable choice.

In a closed system, 45 g of soil amended with both superphosphate and biochar treatments was placed in polyethylene cups. A control treatment without the addition of superphosphate or biochar was also included. Soil moisture was maintained at 14.1% (by weight) throughout the incubation period using 0.005 *M* NaCl to maintain a consistent ionic strength of soil solution. Each treatment was replicated in twenty-one cups. At 0, 1, 2, 4, 6, 9, and 15 weeks of incubation, three cups per treatment were randomly selected to determine available P using 0.5 *M* sodium bicarbonate extraction^29^. Exchangeable K and Ca concentrations were determined using neutral ammonium acetate extraction^23^, whereas soil pH was measured in a 1:1 soil-to-distilled water suspension following 1 h of shaking^23^.

Statistical analyses of available phosphorus (P) and exchangeable potassium (K) data obtained during the incubation experiments were performed using SigmaPlot for Windows (version 15.0.0.13). The analyses included two-way analysis of variance (ANOVA), Shapiro–Wilk test for normality, Brown–Forsythe test for homogeneity of variances, and Fisher’s least significant difference (LSD) test for mean separation across all comparisons. In addition, Spearman’s rank-order correlation was used to evaluate relationships among available P, exchangeable K, exchangeable calcium (Ca), and soil pH.

## Results

### Characteristics of soil and Biochar materials

The chemical analyses of the soil sample revealed that soil exhibited alkaline pH of 8.26 ± 0.05 and low salinity (1.43 ± 0.17 dSm^− 1^). This alkaline soil was characterized with a low CaCO_3_ content of 0.43 ± 0.03%. Besides, low LOI (0.47 ± 0.09%) was quantified indicating a limited OM content^18^.

Moreover, the chemical analyses of the unmodified PF and GB biochars are presented in Table 1. Both PF and GB exhibited alkaline pH of 7.86 and 9.15, respectively. However, high salinity of 14.82 dSm^− 1^ was quantified for PF biochar whereas GB biochar exhibited low salinity of 1.08 dSm^− 1^. Such high salinity that associated with the PF biochar can be mainly ascribed to the high content of soluble K and Ca in PF biochar which were higher by one order of magnitude than the related K and Ca values for GB biochar. Also, the total Na concentration for PF was found to be doubled the associated value for GB (Table 1). The CEC of PF biochar (37.9 cmol_c_ kg^− 1^) was found to be more than twice the CEC of GB biochar (17.6 cmol_c_ kg^− 1^).

**Table 1:** Chemical properties of untreated palm fronds and guava branch biochars.

Soil/Biochar	pH	EC (dSm^− 1^)	CEC (cmol_c_ kg^− 1^)	LOI	*P*		K		Ca		Na	
					Total	Soluble	Total	Soluble	Total	Soluble	Total	Soluble
				%								
Palm frond (PF)	7.86 ± 0.03	14.82 ± 0.16	37.9 ± 2.7	60.8 ± 1.1	0.08 ± 0.00	nd^*^	4.54 ± 0.06	3.45 ± 0.02	1.17 ± 0.02	0.29 ± 0.00	0.61 ± 0.02	0.57 ± 0.00
Guava branches (GB)	9.15 ± 0.03	1.08 ± 0.08	17.6 ± 1.4	56.9 ± 1.6	0.05 ± 0.00	nd	0.29 ± 0.00	0.12 ± 0.00	1.98 ± 0.07	0.01 ± 0.00	0.31 ± 0.00	0.22 ± 0.00

± standard errors; ^*^: not detected;

The chemical properties of the acidified biochar materials are shown in Table 2. The application of different acids decreased the pH value of the original PF and GB significantly. Specifically, the utilization of 0.25 *M* and 0.50 M acetic acid reduced the pH of PF to 5.63 and 5.23, respectively. Whereas pH values of 5.06 and 3.50 were associated with the use of 0.25 M and 0.50 M phosphoric acid, respectively. Likewise for the GB biochar, the use of 0.25 *M* and 0.50 *M* acetic acid reduced the pH to 6.81 and 6.14, respectively. Whereas pH values of 7.38 and 4.16 were associated with the use of 0.25 *M* and 0.50 M phosphoric acid, respectively. The results indicated that no substantial to slight increase of the salinity of PF biochar, whereas the use of acetic acid increased the salinity of GB biochar. Such increase in salinity associated with increasing of soluble Ca and K cations (Table 2). Moreover, acidification of biochar materials caused an increase of the biochar CEC. However, the highest CEC of 55.0 cmol_c_ kg^− 1^ and 30.7 cmol_c_ kg^− 1^ associated with the acidified PF and GB biochar materials, respectively using 0.5 *M* of phosphoric acid.

**Table 2:** Chemical properties of acidified palm fronds and guava branch biochars.

Biochar	Acid concentration (M)	pH	EC (dSm^− 1^)	CEC (cmol_c_ kg^− 1^)	Soluble cations (%)		
					Na	K	Ca
Palm fronds (PF)	Acetic 0.25 *M*	5.63 ± 0.01	14.54 ± 0.31	37.7 ± 1.0	0.59 ± 0.03	3.32 ± 0.16	0.36 ± 0.02
	Acetic 0.50 M	5.23 ± 0.02	15.10 ± 0.20	44.1 ± 1.9	0.54 ± 0.03	3.12 ± 0.18	0.37 ± 0.02
	Phosphoric 0.25 *M*	5.06 ± 0.02	14.55 ± 0.03	43.5 ± 3.3	0.63 ± 0.01	3.53 ± 0.04	0.08 ± 0.00
	Phosphoric 0.50 *M*	3.50 ± 0.02	15.57 ± 0.11	55.0 ± 0.8	0.60 ± 0.01	3.39 ± 0.06	0.08 ± 0.00
Guava branches (GB)	Acetic 0.25 *M*	6.81 ± 0.05	3.14 ± 0.21	17.9 ± 1.1	0.24 ± 0.02	0.18 ± 0.02	0.37 ± 0.02
	Acetic 0.50 M	6.14 ± 0.04	5.37 ± 0.11	20.4 ± 1.2	0.29 ± 0.00	0.22 ± 0.00	0.91 ± 0.03
	Phosphoric 0.25 *M*	7.38 ± 0.16	1.27 ± 0.06	28.6 ± 0.2	0.23 ± 0.00	0.15 ± 0.01	0.01 ± 0.00
	Phosphoric 0.50 *M*	4.16 ± 0.08	2.53 ± 0.14	30.7 ± 1.9	0.26 ± 0.03	0.21 ± 0.02	0.01 ± 0.00

± standard errors.

**Fig. 1:**
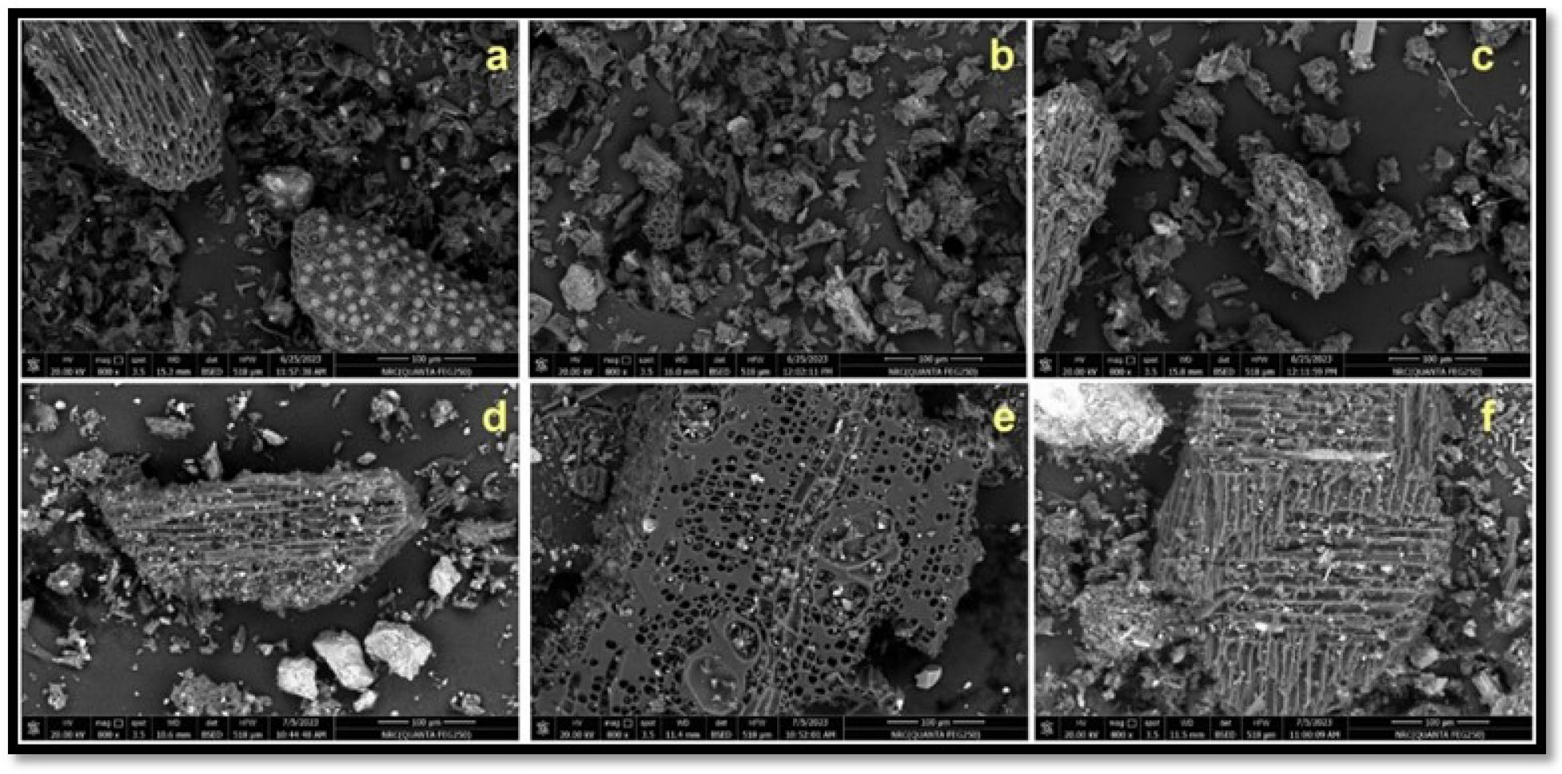
Scanning electron microscope (SEM) images of palm frond-biochar (PF) without acid treatment (**a**), PF-treated with 0.25 *M* acetic acid (**b**), PF-treated with 0.25 *M* phosphoric acid (**c**), and guava branches-biochar (GB) without acid (**d**), GB-treated with 0.25 *M* acetic acid (**e**), GB-treated with 0.25 *M* phosphoric acid (f).

Scanning electron microscope images of the biochar and the acidified materials were shown in Fig. 1. Fibrous structures were observed for the untreated PF and GB biochar materials. The surface morphology for each biochar were distinguished from each other indicating the influence of the different feedstock types (Fig. 1a and d). The microstructure of the unmodified PF biochar exhibited well developed oval-pores that were frequently covered with defined white dots indicating the incidence of mineral phase on the surface of the carbon-lattice. The microstructure of the untreated GB biochar exhibited well developed rectangular holes that were less covered with white dots in comparison with PF biochar. The results of EDX analysis revealed that the main elemental composition (atomic base) of the unmodified PF biochar was 53.90%, 35.14%, 3.10%, 1.18%, 1.36%, 1.05%, and 0.79% for C, O_2_, K, Na, Mg, Si, and Ca, respectively. The respective values for the untreated GB biochar were 55.57%, 38.10%, 0.27%, 1.11%, 0.48%, 0.34%, and 3.75%, respectively. Such results confirm that the dominant cation in the PF and GB biochar was K and Ca, respectively. The surface morphology of the acidified PF with 0.25 *M* acetic acid or 0.25 *M* phosphoric acid indicates the disappearance of the white dots and a decreasing in particles size (Fig. 1b and c). The acidification of GB with 0.25 *M* acetic acid or 0.25 *M* phosphoric acid improved the pores development with minimum influence on particles size (Fig. 1e and f).

**Fig. 2:**
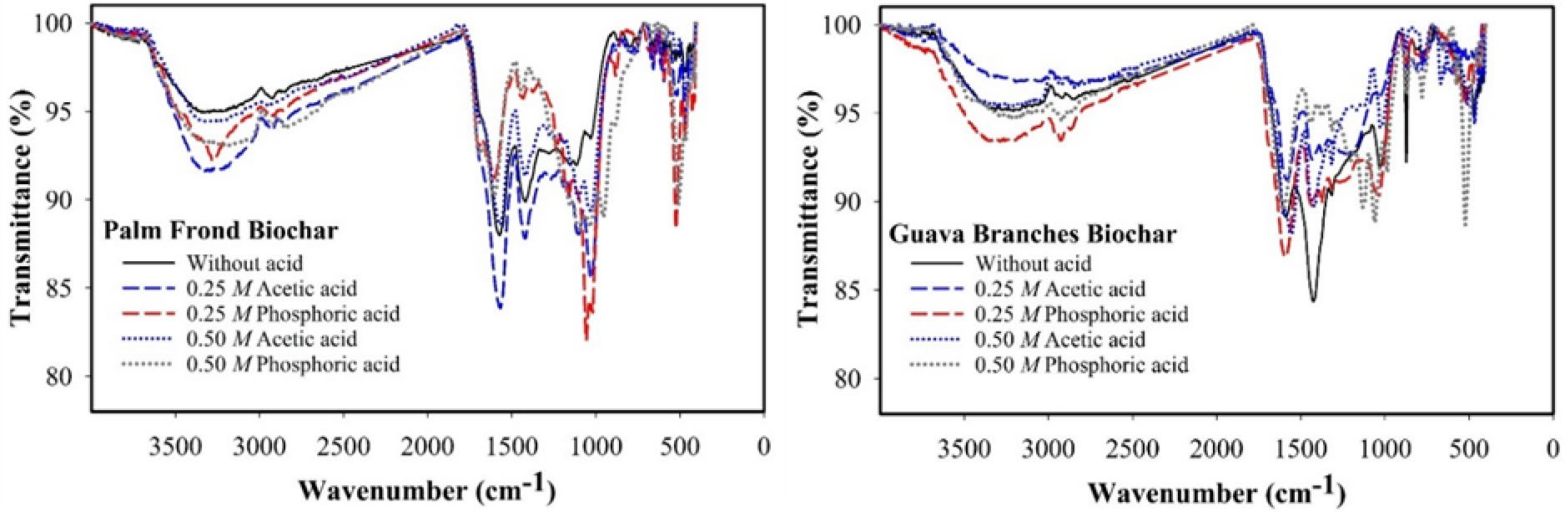
Fourier transform infrared (FTIR) spectroscopy for treated and untreated palm frond-biochar and guava branches-biochar with acetic and phosphoric acids.

Fourier-transform infrared (FTIR) spectra of modified and unmodified PF and GB biochars, modified with acetic and phosphoric acids, are presented in Fig. 2. The detailed assignments of detected bands and their associated wavenumbers are provided in Table S1. In the spectrum of untreated PF, the presence of functional groups such as aliphatic ether, sulfonate or sulfone, aliphatic alcohol, hydroxy compounds, and possibly phosphonic compounds was observed. In contrast, the unmodified GB spectrum indicated the presence of aliphatic alcohol and aliphatic ether functional groups, with possible contributions from organic halogens and hydroxy compounds.

For PF treated with 0.25 *M* acetic acid, the spectrum revealed the occurrence of aliphatic ether or sulfoxide, aliphatic alcohol, and hydroxy compound functional groups. Treatment with 0.50 *M* acetic acid showed bands corresponding to aliphatic alcohol and aliphatic carboxylic acid functional groups. In the case of 0.25 *M* phosphoric acid, the PF spectrum indicated aliphatic ether or sulfonate salt, aliphatic alcohol, and hydroxy functional groups. The 0.50 *M* phosphoric acid-treated PF spectrum revealed more complex functionality, including aliphatic ether, aliphatic amide, organic halogen, ethoxy silane, and aliphatic alcohol groups.

For GB treated with 0.25 *M* acetic acid, the spectrum indicated the presence of aliphatic ether or sulfonate salt, aliphatic alcohol, aliphatic sulfonyl chloride, and organic halogen compounds. The 0.50 *M* acetic acid-treated GB spectrum showed bands corresponding to aliphatic alcohol, aliphatic mercapto groups, and fluorine-containing compounds. In the case of 0.25 *M* phosphoric acid, the GB spectrum revealed the presence of pyridines, aliphatic ether or sulfoxide, aliphatic alcohol, aliphatic thiocompounds, and hydroxy compounds. Finally, the 0.50 *M* phosphoric acid-treated GB spectrum indicated aliphatic amine, aliphatic ether or sulfonate salt, aliphatic alcohol, and organic halogen functional groups (see Table S1 for details).

### Incubation experiments

#### Phosphorus availability status

The concentrations of the available P during the incubation of superphosphate with treated soil are presented in Table 3. In the control soil (without superphosphate or biochar), available P ranged from 27.26 ± 1.40 mg kg^-1^ to 43.55 ± 4.49 mg kg^-1^ over the 15-week incubation period. With superphosphate addition, the range expanded as from 20.46 ± 0.35 mg kg^-1^ to 55.76 ± 2.69 mg kg^-1^. Fixation of P and low availability in alkaline soil are common feature due to high pH and formation of insoluble calcium phosphates. The highest available P (104.02 ± 6.48 mg kg^-1^) was recorded after one week in soil amended with 4.8 Mg ha⁻¹ of phosphoric acid-treated GB biochar. A decline in P availability by week 15 indicates P fixation, observed in both the superphosphate-only treatment and the treatment with 4.8 Mg ha^-1^ of unacidified PF biochar. Overall, available P concentrations declined over time, with an average from 62.42 ± 4.99 mg kg^-1^ at one week to 34.70 ± 2.21 mg kg^-1^ at 15 weeks. Among all treatments, the application of 4.8 Mg ha^-1^ of phosphoric acid-treated GB biochar resulted in the highest mean available P concentration (64.17 ± 9.01 mg kg^-1^) over the incubation period (Table 3).

Statistical analysis of available P during the incubation period revealed a significant difference (*p* ≤ 0.001) among the biochar treatments and incubation durations. A two-way analysis of variance (ANOVA) was conducted after confirming that the data met the assumptions of normality (Shapiro-Wilk test, *p* = 0.160) and homogeneity of variances (Brown-Forsythe test, *p* = 0.001). The ANOVA results indicated statistically significant effects of biochar treatment, incubation period, and their interaction on available P. This result indicate that the impact of biochar treatment depends on the length of the incubation period.

**Fig. 3:**
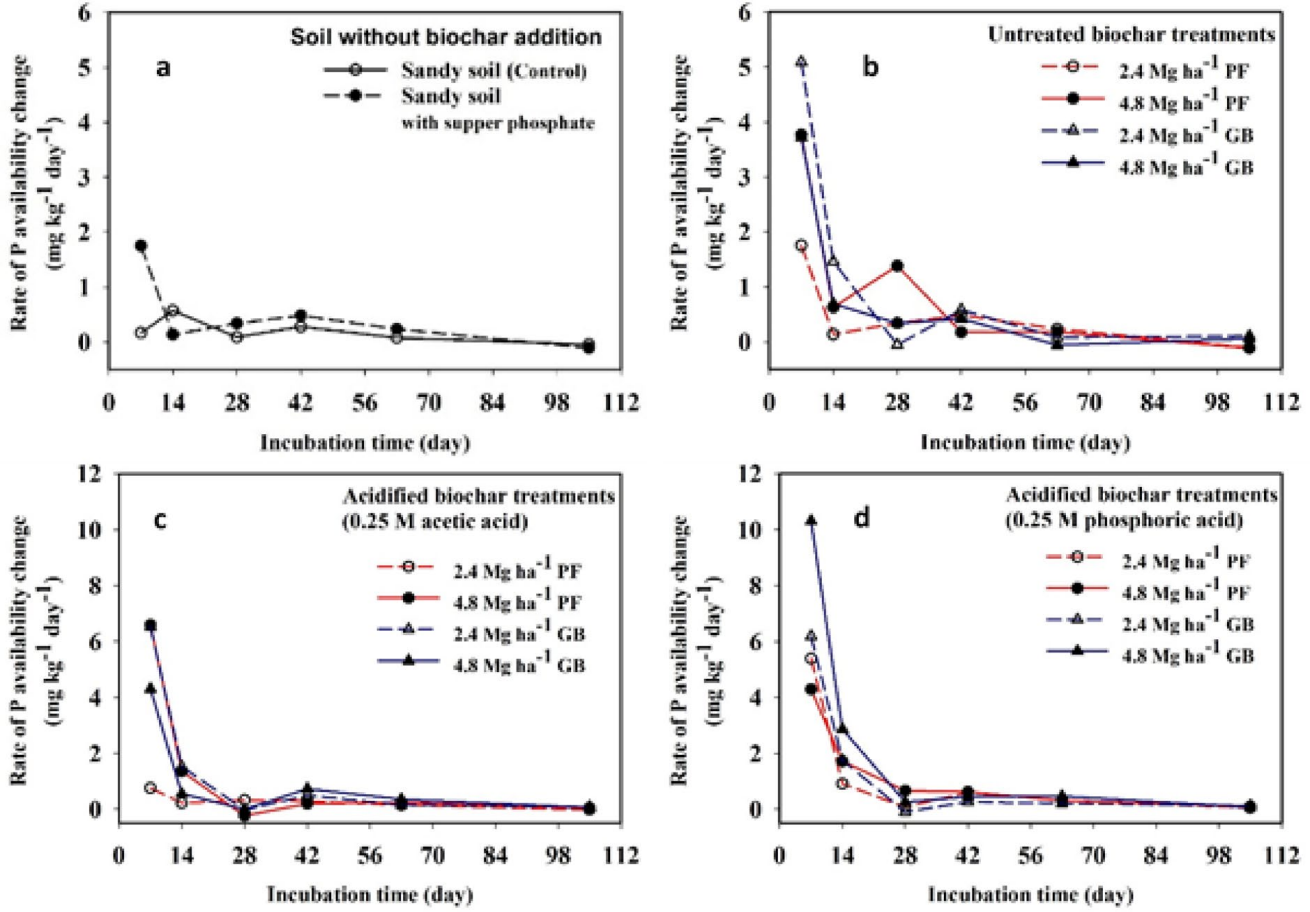
Rate of change in phosphorus (P) availability during the incubation of alkaline sandy soil: (**a**) without biochar, (**b**) with untreated biochar, (**c**) with acetic acid–modified biochar, and (**d**) with phosphoric acid–modified biochar.

For post-hoc analysis, Fisher’s Least Significant Difference (LSD) test was applied at an alpha level of 0.05. The LSD values were 3.00 and 2.02 for treatment and incubation period, respectively (Table 3). Results showed that soil treated with 4.8 Mg ha^-1^ of phosphoric acid-acidified GB biochar had significantly higher P availability compared to all other treatments at both zero and one week of incubation. After 15 weeks, the soil treated with 2.4 Mg ha^-1^ of phosphoric acid-acidified GB biochar exhibited the highest P availability (45.33 ± 1.48 mg kg^-1^), surpassing the 4.8 Mg ha^-1^ treatment. At this point, all treated soils demonstrated significantly higher P availability than the untreated control, except for the treatment with 4.8 Mg ha^-1^ of unacidified PF biochar (Table 3).

On average, all PF biochar treatments resulted in a significant increase in P availability except the 2.4 Mg ha^-1^ doses of both unacidified PF and acetic acid-acidified PF, which showed no significant improvement. For GB biochar treatments, all application rates significantly increased P availability compared to the control. However, no significant differences were observed between low and high application rates—except for the phosphoric acid-acidified GB biochar, where 4.8 Mg ha^-1^ resulted in significantly higher P availability than 2.4 Mg ha^-1^ (Table 3).

The rate of change in P availability during the incubation is shown in Fig. 3. The concentration of the available P (*P*_0_) in the control soil (without superphosphate or biochar) at the start of incubation was used as the reference point for calculating the rate of change (R), as described in Eq. 1:

1 $$\:\text{R}=\frac{{P}_{t}-{P}_{0}}{\text{t}}$$

where R is the rate of change in P availability (mg kg^− 1^ day^− 1^), *P*_t_ is the available P (mg kg^− 1^) after incubation time t (in days).

**Table 3:** Available phosphorus concentrations during the incubation of superphosphate with alkaline sandy soil amended with various Biochar materials over different time periods. Standard errors are shown in parentheses.

Time of incubation (Week)	Soil (control)	Soil with superphosphate	Biochar materials														Average
			Palm frond biochar (PF)								Guava branches biochar (GB)						
			Unacidified			Acidified with acetic acid			Acidified with phosphoric acid		Unacidified		Acidified with acetic acid		Acidified with phosphoric acid		
			*R*_1_^*^	*R*_2_^*^		*R*_1_	*R*_2_		*R*_1_	*R*_2_	*R*_1_	*R*_2_	*R*_1_	*R*_2_	*R*_1_	*R*_2_	
	Available *P* mg kg^− 1^																
Zero	31.85 (2.57)	55.76 (2.69)	47.66 (0.52)		48.39 (2.56)	61.73 (0.30)		54.02 (1.92)	62.67 (2.83)	74.28 (3.35)	62.32 (1.20)	77.94 (2.90)	61.40 (2.33)	66.28 (2.09)	70.98 (0.66)	82.64 (0.67)	61.28 (3.57)
1	32.99 (0.01)	44.10 (2.43)	47.91 (0.96)		58.22 (3.98)	37.17 (6.73)		78.01 (4.18)	69.62 (7.03)	61.86 (2.23)	67.47 (2.41)	57.90 (4.89)	77.51 (4.45)	61.97 (1.16)	75.15 (5.44)	104.02 (6.48)	62.42 (4.99)
2	39.84 (1.89)	33.72 (1.93)	45.95 (1.78)		40.62 (2.91)	34.92 (0.20)		50.86 (0.40)	44.63 (1.53)	55.86 (1.24)	52.30 (1.49)	41.52 (2.59)	53.20 (2.09)	39.65 (1.85)	56.40 (2.87)	71.95 (4.96)	47.25 (2.75)
4	34.37 (3.16)	41.32 (1.64)	34.00 (1.90)		70.51 (1.40)	40.96 (2.12)		25.37 (0.99)	33.99 (0.02)	50.36 (0.85)	30.31 (1.76)	41.27 (1.10)	31.24 (1.60)	31.91 (1.11)	29.12 (0.77)	39.03 (0.43)	38.13 (3.03)
6	43.55 (4.49)	52.07 (1.60)	35.90 (3.42)		39.10 (2.18)	44.12 (1.55)		40.17 (3.72)	57.11 (1.35)	57.80 (3.48)	56.21 (1.77)	49.42 (1.84)	52.59 (2.09)	62.42 (1.61)	42.88 (0.85)	50.66 (0.88)	48.86 (2.15)
9	36.7 (3.41)	46.64 (2.72)	42.88 (1.47)		43.08 (3.49)	41.21 (1.44)		47.43 (3.24)	51.35 (2.99)	51.35 (0.94)	36.80 (0.79)	28.19 (0.20)	41.89 (6.85)	54.22 (2.01)	44.71 (2.23)	61.15 (2.57)	44.83 (2.21)
15	27.26 (1.40)	20.46 (0.35)	27.76 (1.99)		19.78 (1.22)	29.69 (3.30)		37.22 (1.75)	36.54 (1.80)	42.57 (1.46)	42.77 (2.78)	37.87 (0.20)	40.53 (1.45)	38.30 (0.49)	45.33 (1.48)	39.77 (2.69)	34.70 (2.21)
Average	35.22 (2.03)	42.01 (4.50)	40.30 (2.96)		45.67 (6.03)	41.40 (3.84)		47.58 (6.24)	50.84 (5.02)	56.30 (3.79)	49.74 (5.15)	47.73 (6.13)	51.19 (5.78)	50.68 (5.23)	52.08 (6.21)	64.17 (9.01)	

^*^R_1_ and R_2_ are two rates (2.4 and 4.8 Mg ha^− 1^) of the applied biochar or the acidified biochar. Fisher’s least significant differences (LSD, alpha = 0.05) are 3.00and 2.02 for biochar treatment and incubation time factors, respectively.

In the control soil, P availability showed minimal change during the first six weeks, followed by a negative rate of -0.04 mg kg⁻¹ day⁻¹ after nine weeks, indicating P fixation. In the soil treated with superphosphate only (no biochar), the highest positive change was observed after one week (1.75 mg kg^-1^ day^-1^), with limited changes until six weeks, followed by P fixation at a rate of – 0.11 mg kg^-1^ day^-1^ after 15 weeks (105 days).

When unacidified biochar was applied, 2.4 and 4.8 Mg ha⁻¹ of GB biochar yielded rates of 5.09 and 3.72 mg kg^-1^day^-1^, respectively, after one week. Similarly, 2.4 and 4.8 Mg ha^-1^ of PF biochar produced rates of 2.30 and 3.77 mg kg^-1^ day^-1^, respectively. No P fixation was observed with untreated GB biochar, while negative rates—indicating fixation—were recorded for PF biochar after 15 weeks.

With acidified biochar using acetic acid, P availability improved: applying 2.4 and 4.8 Mg ha^-1^ of acidified GB and PF biochars resulted in rates of 6.52 and 6.54 mg kg^-1^ day^-1^, respectively, after one week, with no fixation observed after 15 weeks. Notably, the use of phosphoric acid–acidified biochar resulted in the highest increase in P availability with a rate of 10.31 mg kg^-1^ day^-1^, observed one week after applying 4.8 Mg ha^-1^ of GB biochar, with no fixation after 15 weeks (Fig. 3).

#### Potassium availability status

Changes in exchangeable K during the incubation experiment are presented in Table 4. The results demonstrate the influence of biochar content on K availability in the incubated soils. Notably, PF biochar contained higher amounts of total and soluble K compared to GB biochar (Table 1), resulting in increased exchangeable K levels in soils treated with PF biochar. At the beginning of the incubation (day 0), the untreated soil exhibited the lowest exchangeable K concentration, at 44.22 ± 3.95 mg kg⁻¹. Across all incubation periods, the average exchangeable K was 62.82 ± 2.10 mg kg⁻¹ for the control soil and 69.06 ± 2.29 mg kg⁻¹ for soil treated with superphosphate alone (Table 4).

The exchangeable K data passed the Shapiro-Wilk normality test (*p* = 0.740) and the Brown-Forsythe equal variance test (*p* = 0.031). ANOVA results indicated a statistically significant interaction between biochar treatment and incubation time (*p* = 0.001). According to the Fisher LSD test for multiple comparisons (α = 0.05), the least significant differences were 4.13 for biochar treatment and 2.86 for incubation time (Table 4). These results confirmed significant differences between the effects of PF and GB biochars.

**Table 4:** Concentrations of exchangeable potassium during the incubation of superphosphate with alkaline sandy soil treated with various Biochar materials over different time periods. Standard errors are shown in parentheses.

Time (Week)	Soil (control)	Soil with superphosphate	Biochar materials												Average
			Palm frond biochar (PF)						Guava branches biochar (GB)						
			*R*_1_	*R*_2_	*R*_1_	*R*_2_	*R*_1_	*R*_2_	*R*_1_	*R*_2_	*R*_1_	*R*_2_	*R*_1_	*R*_2_	
			Unacidified		Acidified with acetic acid		Acidified with phosphoric acid		Unacidified		Acidified with acetic acid		Acidified with phosphoric acid		
	Exchangeable K mg kg^− 1^														
Zero	44.22 (3.95)	90.61 (4.97)	84.07 (2.83)	110.61 (11.88)	73.92 (4.04)	125.38 (4.63)	109.7 (0.83)	101.97 (3.02)	50.74 (1.70)	67.05 (3.53)	70.28 (2.52)	57.45 (4.10)	65.50 (0.33)	58.20 (3.19)	79.26 (1.52)
1	74.14 (1.16)	85.9 (3.41)	114.11 (0.16)	153.00 (13.76)	118.96 (1.54)	116.33 (3.51)	114.32 (7.47)	108.75 (4.53)	97.40 (2.70)	87.30 (3.43)	84.99 (3.85)	71.81 (8.18)	73.76 (0.10)	66.15 (3.69)	97.64 (1.65)
2	69.25 (4.41)	61.53 (1.12)	92.73 (1.49)	79.66 (9.79)	86.05 (3.91)	108.88 (2.94)	77.53 (2.37)	95.28 (0.90)	59.69 (3.31)	52.79 (2.01)	70.34 (3.47)	70.94 (2.39)	68.51 (5.69)	72.85 (2.22)	76.14 (1.45)
4	75.14 (3.64)	69.54 (6.29)	88.48 (3.62)	110.20 (3.61)	86.95 (2.87)	119.86 (3.02)	92.07 (3.06)	129.65 (3.75)	61.5 (3.31)	64.47 (1.98)	70.17 (3.61)	57.22 (4.10)	64.87 (3.72)	66.85 (3.26)	82.64 (1.53)
6	68.53 (4.23)	57.18 (3.02)	78.93 (0.85)	90.27 (2.85)	69.20 (4.67)	82.63 (1.69)	73.04 (0.53)	94.73 (4.57)	54.82 (2.97)	53.46 (2.23)	60.29 (1.83)	57.4 (2.31)	63.6 (2.24)	55.97 (2.49)	68.57 (1.19)
9	51.39 (1.85)	60.98 (2.92)	70.83 (1.43)	92.34 (3.28)	74.19 (7.64)	87.92 (5.29)	63.78 (1.01)	97.62 (2.81)	51.56 (0.82)	46.16 (0.96)	44.96 (1.22)	54.82 (0.60)	48.88 (1.23)	54.56 (3.02)	64.29 (1.10)
15	57.05 (3.92)	57.67 (5.94)	76.04 (2.93)	92.31 (3.75)	68.83 (4.30)	88.98 (4.67)	71.49 (2.63)	91.08 (1.09)	52.53 (2.96)	63.53 (3.07)	55.46 (1.58)	51.36 (3.40)	54.92 (2.84)	54.95 (2.19)	66.87 (1.40)
Average	62.82 (2.10)	69.06 (2.29)	86.45 (1.17)	104.05 (3.86)	82.59 (2.66)	104.28 (2.24)	85.99 (1.53)	102.73 (1.87)	61.18 (1.61)	62.11 (1.50)	65.21 (1.60)	60.14 (2.08)	62.86 (1.50)	61.36 (1.81)	

^*^ R_1_ and R_2_ are two rates (2.4 and 4.8 Mg ha^− 1^) of the applied biochar or the acidified biochar. Fisher’s least significant differences (LSD, alpha = 0.05) are 4.13 and 2.86 for biochar treatment and incubation time factors, respectively.

For PF biochar, a significant increase in exchangeable K was observed with the application rate of 4.8 Mg ha⁻¹ compared to 2.4 Mg ha⁻¹, based on treatment averages. However, no significant differences were found between the control and soils treated with GB biochar (both acidified and unacidified). Based on the average of each treatment, soils treated with GB biochar (acidified and unacidified) exhibited significantly lower exchangeable K than those treated with superphosphate/no biochar (Table 4). Notably, the effects of biochar treatments varied over time, as indicated by fluctuations in exchangeable K and the significant interaction between treatments and incubation time.

#### Influence of pH and the exchangeable Ca on P and K availability

The potential influence of soil pH and exchangeable Ca variations on P and K availability was evaluated. During incubation, the pH of the control soil ranged from 7.72 to 8.61, while the soil treated with superphosphate (without biochar) exhibited a pH range of 7.54 to 8.72. In both cases, the lowest pH values were observed after 15 weeks of incubation. Besides, exchangeable Ca in the control soil ranged from 410.8 to 735.7 mg kg⁻¹, with an average of 514.1 mg kg⁻¹. In the superphosphate-treated soil (without biochar), exchangeable Ca ranged from 371.9 to 749.2 mg kg⁻¹, averaging 534.5 mg kg⁻¹. Similar to the pH trend, the lowest exchangeable Ca concentrations in both treatments were also observed at the 15-week.

**Fig. 4:**
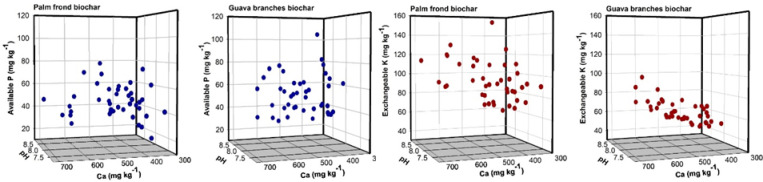
3D scatter plots showing variations in phosphorus (P) availability and exchangeable potassium (K) in relation to soil pH and exchangeable calcium (Ca) during the incubation of superphosphate with alkaline sandy soil amended with palm frond and guava branch biochars.

The 3D scatter plots in Fig. 4 illustrate the variability of P availability and the exchangeable K during soil incubation with superphosphate and different biochar amendments. Specifically, soil pH and exchangeable Ca were plotted on the x- and y-axes, respectively, as independent variables. The results demonstrate that soil pH varied according to the type of biochar applied (PF or GB), across both unacidified and acidified treatments. Soil pH ranged from 7.28 to 8.67 for PF-biochar-treated soils and from 7.61 to 8.75 for GB-biochar-treated soils. Exchangeable Ca ranged from 334.6 to 697.8 mg kg⁻¹ for PF-biochar and from 372.7 to 635.2 mg kg⁻¹ for GB-biochar.

For PF-biochar-treated soils, the plots in Fig. 4 depict that P availability exhibited minimal dependence on either soil pH or exchangeable Ca. High levels of available P were observed at both pH values below 7.5 and above 8.1, with corresponding exchangeable Ca levels between 400 and 600 mg kg⁻¹. In contrast, for GB-biochar-treated soils, the highest P availability occurred within a narrower pH range of 7.8 to 8.2, with no clear relationship to exchangeable Ca. On the other hand, exchangeable K displayed a more pronounced association with both soil pH and exchangeable Ca, particularly in the GB-biochar treatments.

Furthermore, statistical relationships among available P, exchangeable K, exchangeable Ca, and soil pH were assessed using the Spearman rank-order correlation test. The resulting correlation coefficients and p-values are presented in Table 5. A p-value below 0.05 indicates a statistically significant correlation, with negative and positive coefficients representing inverse and direct relationships, respectively.

For PF-biochar–incubated soil, no significant correlations were observed between available P and soil pH, available P and exchangeable Ca, or exchangeable K and exchangeable Ca. A moderate significant correlation was found between pH and exchangeable Ca, while weak but significant correlations were noted between pH and exchangeable K, and between available P and exchangeable K. In the case of GB-biochar–incubated soil, no significant correlations were observed between available P and soil pH, available P and exchangeable Ca, or soil pH and exchangeable K. However, moderate significant correlations were found between pH and exchangeable Ca, and between exchangeable K and exchangeable Ca. A weak significant correlation was also observed between available P and exchangeable K.

**Table 5:** Spearman rank-order correlation coefficients among available P, exchangeable K, pH, and exchangeable Ca during the incubation of alkaline sandy soil amended with various Biochar materials. P-values are presented in parentheses; correlations are considered significant at *P* < 0.05.

	Available *P*	pH	Exchangeable Ca
Palm frond biochar (PF)			
pH	0.036 (0.8220)		
Exchangeable Ca	– 0.018 (0.9100)	0.509 (0.0006)	
Exchangeable K	0.397 (0.0096)	0.307 (0.0480)	0.279 (0.0737)
Guava branches biochar (GB)			
pH	– 0.267 (0.0876)		
Exchangeable Ca	– 0.087 (0.5830)	0.527 (0.0004)	
Exchangeable K	0.387 (0.0115)	0.239 (0.1260)	0.574 (0.0001)

## Discussions

### Characterization of Biochar materials

Variations in biochar properties are expected when different feedstocks are utilized. Pradhan et al.^30^ reported a pH range of 7.2 to 11.6, attributed to differences in feedstock materials and pyrolysis temperatures. In the current study, both PF and GB exhibited an alkaline reaction; however, they differed in their morphological and chemical characteristics (see Table 1). These findings align with previous studies; Abdel-Hamied and El-Shiekha^31^ reported an alkaline pH range of 8.12 to 8.25 for guava tree-derived biochar, while Badawi^32^ found a pH range of 6.8 to 8.9 for palm frond-derived biochar. Sizirici et al.^33^ reported a pH of 9.2, 9.3, and 9.9 for palm frond-derived biochar that was pyrolyzed at 400, 500 and 600 °C, respectively. Moreover, PF biochar was notable for its high CEC and K content, indicating strong potential for agricultural applications. As well, GB biochar was favorably characterized by lower salinity and higher calcium (Ca) content compared to PF. The biochar is inherently a complex material, comprising both organic carbon and inorganic salts. Our chemical and EDX analyses suggest that the elevated salinity observed in PF biochar is primarily due to its high levels of soluble and total K, indicating significant K enrichment. This is supported by Som et al.^34^, who reported that K constituted 87% of the total cation content in palm frond-derived biochar. As such, PF biochar may serve as an important source of K nutrition in sandy soils. In contrast, GB biochar contains low total K and, therefore, cannot be considered a viable K source (see Table 1). Consistent with our findings, Abdel-Hamied and El-Shiekha^31^ reported a low available K content of less than 0.03% in guava tree-derived biochar.

Acidification of the biochar materials effectively reduced their pH and enhanced the CEC. The reduction in pH can be attributed to chemical reactions that lead to the protonation of functional groups and the formation of aliphatic and phenolic hydroxyl groups. For example, acetic acid, a weak organic acid, introduces carboxyl and hydroxyl groups, while phosphoric acid, a strong inorganic acid, introduces phosphate groups, both contributing to biochar acidity. Moreover, the negative surface charge of the treated and untreated biochar is likely due to the presence of reactive functional groups such as aliphatic alcohol, hydroxy and carboxylic acid. Lago et al.^35^ reported a wide range for CEC of different feedstock-derived biochar materials, spanning from 4.0 to 150 cmol_c_ kg^− 1^, where the high CEC values attributed to the presence of phenolic and carboxylic functional groups. Notably, a significant increase in CEC was achieved with the application of 0.5 M phosphoric acid; however, P-related functional groups were not distinctly detected in FTIR spectra. The ATR-FTIR provide limited detectability of P-related functional groups at low P concentration due to the peaks overlap in the fingerprint region^36^. Moreover, acidification had minimal impact on the salinity of PF biochar, whereas treatments with acetic acid or 0.5 M phosphoric acid led to a twofold increase in the salinity of GB biochar. This increase is likely attributed to the solubilizing action of acetic and phosphoric acids, particularly under acidic conditions, which enhance the release of soluble salts and inorganic constituents. Similarly, Chang et al.^37^ reported that sulfuric and nitric acids effectively dissolved the inorganic fraction of biochar.

Characterization of PF and GB biochars revealed their high pH, which could further exacerbate alkalinity in the studied sandy soil. Acidifying both biochars was therefore a practical strategy to improve nutrient availability. A lower acid concentration (0.25 M) was preferable, as it met sustainable soil management objectives and avoided the salinity increase observed with 0.50 M phosphoric acid. These findings emphasize the importance of thorough biochar characterization to guide its effective use as a soil amendment.

### Influence of Biochar on P and K availability

The incubation experiments revealed diverse effects of biochar and modified biochar materials on P and K availability in alkaline sandy soil. Application of superphosphate alone led to an immediate increase in P availability by 23.91 mg kg⁻¹ compared to the control. Notably, greater initial increases in P availability were observed with GB biochars than with PF biochars, especially those modified with phosphoric acid. In contrast, unacidified PF biochars initially reduced P availability due to a fixation effect, although availability later surpassed that of the treatment without biochar. Elbana et al.^9^ similarly reported a substantial rise in available P after one week of incubating alkaline sandy soil with low-grade phosphate rock, followed by minimal change over the subsequent 12 weeks. The immediate increase in P from superphosphate is likely due to its chemically available form, which readily dissolves and transforms into labile soil P fractions^38^. An increase in P availability that was associated with the addition of acidified biochars can be ascribed to the neutralize alkalinity effect and the formation of acidic functional groups on biochar surfaces^39^.

The results indicate that the influence of acidified or nonacidified biochar on P availability depends on the biochar specification. In agreement with our results regarding the fluctuation of P availability during the incubation, Qayyum et al.^40^ reported that incubation of a silt loam alkaline soil with an acidified rice husk-biochar exhibited higher P availability than that soil treated with the nonacidified biochar with observation of inconsistency during 60 days of incubation, whereas a steady decrease in P availability was observed when nonacidified wheat straw-biochar was applied. In the current study, using high rate of the phosphoric acid acidified-GB biochar maintained consistently high P availability throughout the incubation period, whereas the low rate exhibited inconsistency. Thus, the acidified-GB biochar with phosphoric acid can effectively facilitate the slow release of P in soil. Zhang et al.^15^ highlighted the potential of biochar to gradually supply P to plants through a dynamic equilibrium process.

Numerical analysis of the calculated rates of change in P availability revealed a rapid decline within the first two weeks, followed by P fixation after six weeks of incubation when superphosphate was applied without biochar. In contrast, the use of acidified biochar markedly enhanced P release into the soil after just one week and significantly reduced P fixation over the 15-week incubation period. Notably, the highest rate of P release was observed with phosphoric acid–acidified GB biochar (see Fig. 3). Similarly, Li et al.^10^ reported a sharp drop in P availability during the first two weeks of incubation, from 281.54 mg P m⁻² to 109.22 mg P m⁻², when soil was treated with triple superphosphate. However, P availability remained significantly higher when acidic P-laden biochar was applied. Also, Khalafalla et al.^41^ found that P release rates in sandy alkaline soil amended with acidified fish waste biochar ranged from 1.77 to 2.01 mg kg⁻¹ day⁻¹ after two days of incubation, decreasing to 0.14–0.20 mg kg⁻¹ day⁻¹ after 100 days.

The statistical analysis showed no significant correlation between the available P and either soil pH or the exchangeable Ca. This lack of association may be attributed to the relatively narrow pH range (7.28 to 8.75) observed during the incubation period. Consistent with these findings, Qayyum et al.^40^ reported a limited reduction in soil alkalinity in silt loam soil amended with acidified biochar derived from wheat straw, rice husk, and sugarcane waste. Similarly, Elbasiouny et al.^6^ found that Olsen extractable P was significantly correlated with Mg, K, CEC, Fe, Al, and silt contents, but not significantly correlated with Ca, Na, or soil pH.

Chemical analysis revealed that PF biochar is enriched with K. Biochar produced from low-lignin feedstock typically contains higher K concentrations compared to wood-derived biochar^14,42^. This finding highlights the potential of PF biochar as a source of K fertilizer. Angst and Sohi^20^ estimated that applying 20 t ha⁻¹ of hardwood biochar derived from *Acer pseudoplatanus* could supply 20–50 kg K ha⁻¹, depending on the particle size. Increasing the rate of biochar application has been shown to enhance plant K uptake, though the extent of this increase varies with soil and crop type^21^.

The results indicated a significant association between available P and exchangeable K, which can be attributed to the positive effects of biochar additions on both nutrients. However, the relationship between exchangeable K and either soil pH or exchangeable Ca varied depending on the type of biochar applied. Specifically, a significant correlation between exchangeable K and soil pH was observed with PF biochar, but not with GB biochar. Conversely, a significant correlation between exchangeable K and exchangeable Ca was found in soils treated with GB biochar, but not with PF. This variation is likely due to differences in the total K content of the biochars, as PF contains approximately an order of magnitude more K than GB (see Table 1).

In the case of incubation with acidified or unacidified PF biochar, the dominance of K combined with relatively low Ca content, likely promoted the preferential occupation of exchange sites by K. In contrast, both acidified and unacidified GB biochars exhibited lower K content and relatively higher Ca content. Thus, the greater selectivity for K on PF-treated soils may be governed by cation stoichiometry, as described by Sparks^43^. Furthermore, the high CEC of the biochars influenced K exchangeability in the soil, taking into account both the infinitely high K-selective sites and the non-specific exchange sites^44^. Our findings showed that PF biochar, particularly in its acidified form, exhibited higher CEC values (see Tables 1 and 2), further supporting its enhanced K retention and exchange behavior.

While this study provides encouraging evidence for the use of acidified biochars to enhance P and K availability in alkaline sandy soils, several limitations should be acknowledged. The results are based on short-term, controlled incubation experiments involving a single soil type, and may not fully capture biochar behavior under field conditions or in other soil environments, such as calcareous or clay-rich soils. Additionally, plant response data were not included in this study but are currently being evaluated through ongoing field trials conducted as part of the broader research project. The long-term stability and environmental impacts of acidified biochars also warrant further investigation, particularly with respect to nutrient leaching, shifts in microbial communities, and the potential accumulation of salts. In the case of PF biochar, its high salinity—primarily due to elevated K content—could pose a risk of salt build-up or sodium-related hazards under certain conditions. Therefore, careful monitoring of soil salinity and appropriate management of application rates are recommended to prevent potential land degradation.

## Conclusions

Acid modification of PF and GB biochars reduced their pH, with phosphoric acid notably increasing CEC. In alkaline sandy soil, phosphoric acid–treated GB biochar significantly enhanced P availability, supporting our hypothesis that acidification can improve nutrient release compared to unmodified forms. Both acidified and unacidified PF biochars increased exchangeable K, particularly at the 4.8 Mg ha⁻¹ rate, indicating that PF biochar can serve as a reliable K source regardless of modification. These findings confirm that acid-modified biochar, especially phosphoric acid–treated GB, can more effectively supply P, while PF biochar can address K limitations in alkaline sandy soils. Selecting the acid type based on the target nutrient is therefore key to maximizing biochar’s agronomic benefits. Field-scale studies are needed to validate these outcomes under practical farming conditions.

## Supplementary Information

Below is the link to the electronic supplementary material.

Supplementary Material 1

## Data Availability

The datasets used or analyzed during the current study are available from the corresponding author upon reasonable request.

## Abbreviations

ANOVA: analysis of variance
ATR-FTIR: attenuated total reflectance fourier-transform infrared (ATR-FTIR)
C: carbon
Ca: calcium
CaCO₃: calcium carbonate
CEC: cation exchange capacity
EC: electrical conductivity
EDX: energy-dispersive X-ray spectroscopy
FEG: field emission gun
FTIR: fourier-transform infrared
GB: guava branch
K: potassium
LOI: loss-on-ignition
LSD: least significant difference
Mg: magnesium
Na: sodium
O_2_: Oxygen
P: phosphorus
P_0_: available p in the control soil at the start of incubation
PF: palm frond
P_t_: the available P at certain incubation time
R: the rate of change in P availability
SDGs: sustainable development goals
SEM: scanning electron microscopy
Si: silicon
SOC: soil organic carbon
t: time
