# Publisher Correction: Influence of acidified-biochar on phosphorus and potassium availability in alkaline sandy soil

**DOI:** 10.1038/s41598-025-24089-2

**Published:** 2025-10-15

**Authors:** Tamer A. Elbana, Noura Bakr, Sahar A. Shahin, Nahed A. A. Azab, Soad M. El-Ashry

**Affiliations:** Soils and Water Use Department, Agricultural and Biological Research Institute, National Research Centre, Cairo, Egypt

Correction to: *Scientific Reports* 10.1038/s41598-025-16247-3, published online 20 August 2025

The original version of this Article contained errors in the order of the figures, where Figures 1, 2 and 3 were incorrectly published as Figures 3, 1 and 2, respectively. As a result, the figure legends did not correspond to the correct figure images. The figures and their accompanying legends were in the correct order in the original manuscript.

The original Figure 1, 2 and 3 and their accompanying legends appear below.

**Fig. 1:**
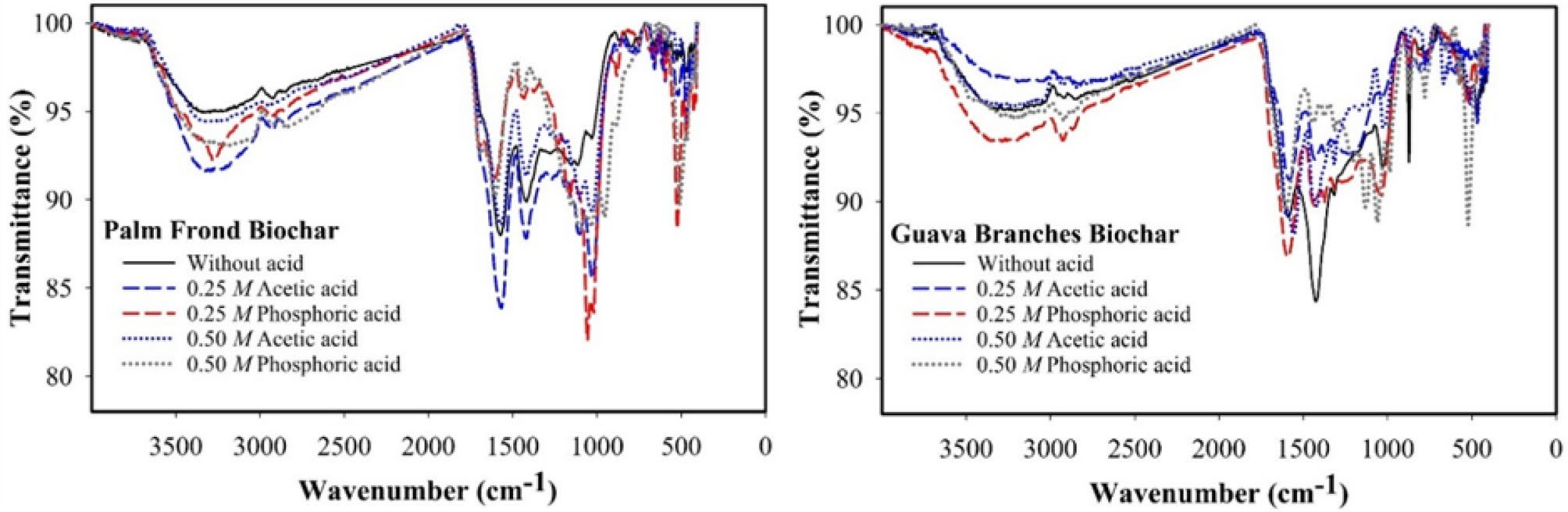
Scanning electron microscope (SEM) images of palm frond-biochar (PF) without acid treatment (**a**), PF-treated with 0.25 *M* acetic acid (**b**), PF-treated with 0.25 *M* phosphoric acid (**c**), and guava branches-biochar (GB) without acid (**d**), GB-treated with 0.25 *M* acetic acid (**e**), GB-treated with 0.25 *M* phosphoric acid (f).

**Fig. 2:**
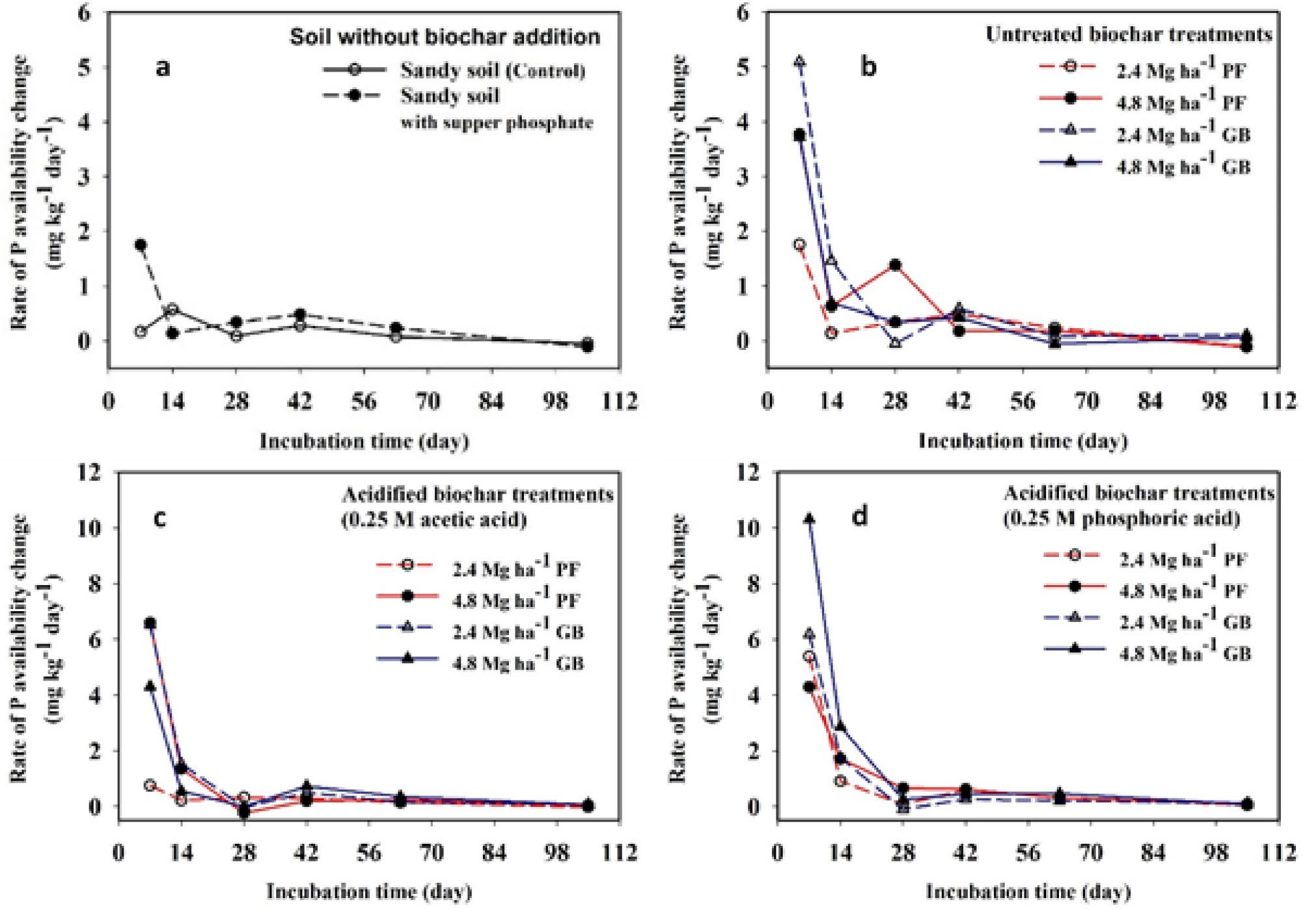
Fourier transform infrared (FTIR) spectroscopy for treated and untreated palm frond-biochar and guava branches-biochar with acetic and phosphoric acids.

**Fig. 3:**
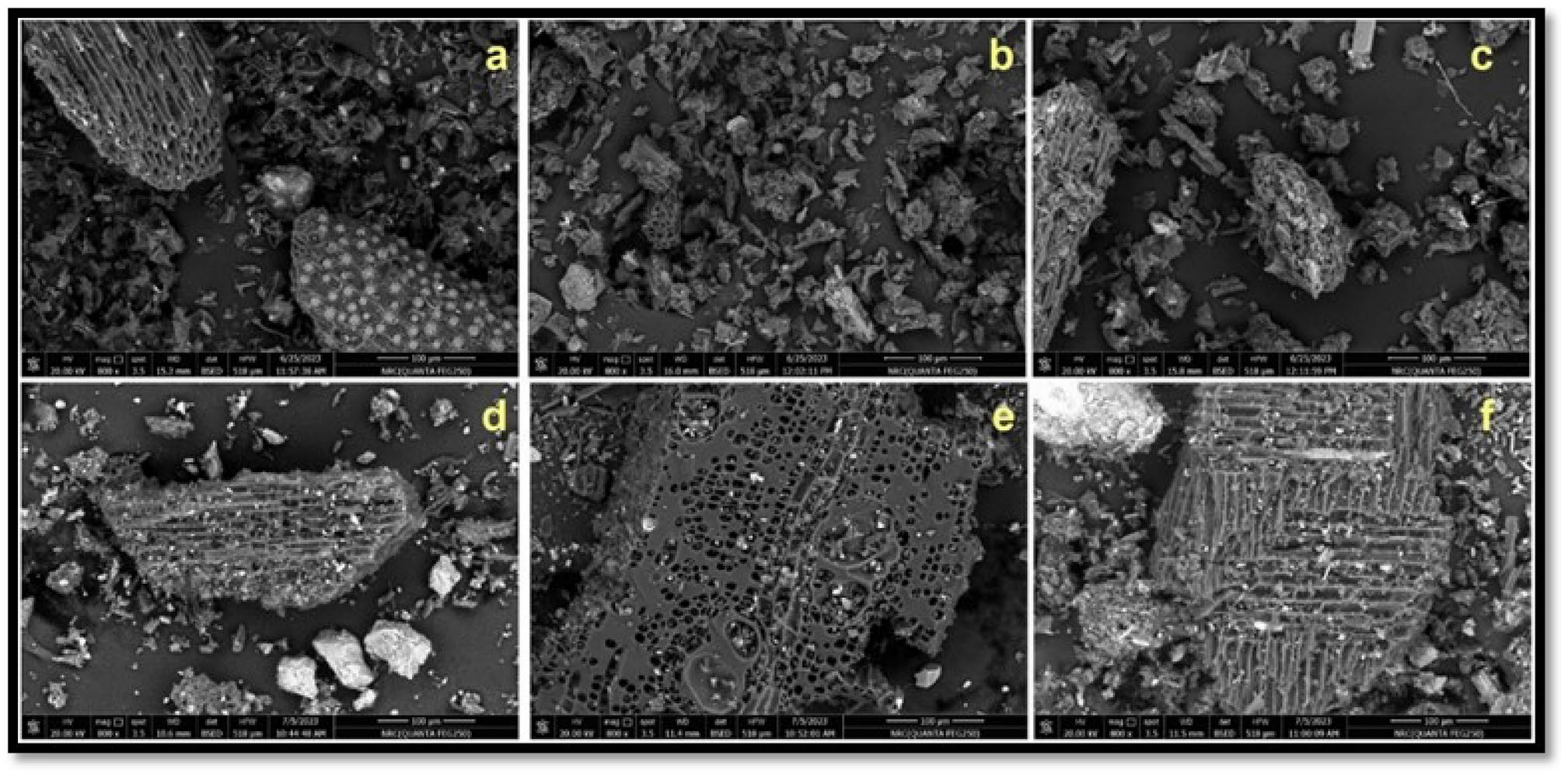
Rate of change in phosphorus (P) availability during the incubation of alkaline sandy soil: (**a**) without biochar, (**b**) with untreated biochar, (**c**) with acetic acid–modified biochar, and (**d**) with phosphoric acid–modified biochar.

The original Article has been corrected.

