# Supplementary material for: Influence of acidified-biochar on phosphorus and potassium availability in alkaline sandy soil
Source: Sci Rep. 2025 Aug 20;15:30504. doi: 10.1038/s41598-025-16247-3 (PMC12368130; doi:10.1038/s41598-025-16247-3)
Supplement: Supplementary file 1 — Supplementary Material 1 [file 41598_2025_16247_MOESM1_ESM.pdf]

Supplementary material

For

Influence of acidified-biochar on phosphorus and potassium availability in alkaline sandy soil

Tamer A Elbana* Noura Bakr Sahar A Shahin Nahed A A Azab Soad M El-Ashry

Soils and Water Use Department, Agricultural and Biological Research Institute, National Research Centre, Cairo, Egypt

* Corresponding author, E-mail: tamerelbana@yahoo.com; ta.elhammed@nrc.sci.eg

Table S1. Detected bands and assigned functional groups from Fourier-transform infrared (FTIR) spectra of acidified and unacidified palm frond (PF) and guava branch (GB) biochars.

Biochar	Defined band	Wavenumber (cm^{-1})	Anticipated functional groups
Untreated PF	▪ C-H stretch- alkyl<br>▪ C-H bend- CH_2<br>▪ SO_2, sulfoxy stretch<br>▪ C-O stretch<br>▪ O-H stretch<br>▪ S-H stretch,<br>▪ P=O stretch	▪ 3000-2850<br>▪ 1470-1415<br>▪ 1325-1275, 1205-1160, 1150-1115, and 1065-1030<br>▪ 1125-1000<br>▪ 3450-3225<br>▪ 2565-2535<br>▪ 1305-1260	▪ Aliphatic ether or sulfonate or sulfone, aliphatic alcohol, hydroxy compound, and possibly phosphonic compound
Untreated GB	▪ C-H stretch- alkyl<br>▪ C-H bend- CH_2/CH_3<br>▪ C-O stretch<br>▪ O-H stretch<br>▪ O-H bend<br>▪ CH3 bend	▪ 2975-2845<br>▪ 1475-1415<br>▪ 1150-1070 and 1085-1000<br>▪ 3450-3225<br>▪ 1400-1300<br>▪ 1450-1420	▪ Aliphatic alcohol and aliphatic ether, organic halogen and hydroxy compound
Acidified PF with 0.25 M acetic	▪ C-H stretch- alkyl<br>▪ C-S=O sulfoxy stretch<br>▪ C-O stretch<br>▪ C-H bend- CH_2/CH_3<br>▪ C-H bend- CH_2/C-S<br>▪ S-H stretch<br>▪ O-H stretch	▪ 3000-2855<br>▪ 1060-1020<br>▪ 1120-1020<br>▪ 1475-1415<br>▪ 1305-1265 and 1450-1410<br>▪ 2565-2535<br>▪ 3430-3340	▪ Aliphatic ether or sulfoxide, aliphatic alcohol, and hydroxy compound
Acidified PF with 0.50 M acetic	▪ C-H stretch- alkyl<br>▪ C-H bend- CH_2/CH_3<br>▪ C-O stretch<br>▪ C=O stretch<br>▪ C-O bend<br>▪ O-H bend<br>▪ O-H bonded-acid<br>▪ O-H stretch	▪ 2975-2845 and 3120-2855<br>▪ 1475-1415 and 1450-1380<br>▪ 1085-1000<br>▪ 1730-1680<br>▪ 500-400<br>▪ 1400-1300<br>▪ 2710-2580<br>▪ 3430-3340	▪ Aliphatic alcohol and aliphatic carboxylic acid
Acidified PF with 0.25 M phosphoric acid	▪ C-H stretch- alkyl<br>▪ C-H bend- CH_2<br>▪ SO_2 sulfoxy stretch<br>▪ C-F bend	▪ 3000-2855<br>▪ 1470-1415<br>▪ 1065-1030 and 1205-1160<br>▪ 520-400	▪ Aliphatic ether or sulfonate salt, aliphatic alcohol, and hydroxy compound

	▪ C-F stretch<br>▪ C-H bend<br>▪ C-O stretch<br>▪ O-H bend<br>▪ O-H stretch	▪ 1090-1020<br>▪ 1390-1340<br>▪ 1070-1020<br>▪ 1400-1300<br>▪ 3450-3300	
Acidified PF with 0.50 M phosphoric acid	▪ C-H stretch- alkyl<br>▪ CH_3 bend<br>▪ C-O stretch<br>▪ C-N stretch<br>▪ NH bend<br>▪ C=O stretch<br>▪ N-H stretch<br>▪ C-F stretch<br>▪ C-F bend<br>▪ C-H bend<br>▪ Si-O-C stretch<br>▪ C-N Stretch/C-C stretch<br>▪ C-H bend<br>▪ O-H bend<br>▪ O-H stretch	▪ 3000-2820<br>▪ 1480-1435 and 1450-1420<br>▪ 1150-1070 and 1070-1020<br>▪ 1420-1400<br>▪ 1620-1590<br>▪ 1700-1670<br>▪ 3430-3370 and 3520-3475<br>▪ 1090-1020<br>▪ 520-400<br>▪ 1390-1340<br>▪ 1100-1070 and 990-945<br>▪ 1150-1050<br>▪ 1460-1410<br>▪ 1400-1300<br>▪ 3450-3300	▪ Aliphatic ether, aliphatic amide, organic halogen, ethoxy silane, and aliphatic alcohol
Acidified GB with 0.25 M acetic	▪ C-H stretch- alkyl<br>▪ C-H bend- CH_2/CH_3<br>▪ SO_2 sulfoxy stretch<br>▪ SO_2 sulfoxy bending<br>▪ S-Cl stretch<br>▪ C-S stretch<br>▪ C-O stretch<br>▪ O-H bend<br>▪ O-H stretch<br>▪ C-F stretch<br>▪ C-F bend	▪ 3000-2855<br>▪ 1470-1415<br>▪ 1065-1030, 1190-1160, 1205-1160, and 1390-1360<br>▪ 550-530 and 590-550<br>▪ 430-400<br>▪ 760-660<br>▪ 1085-1000 and 1210-1100<br>▪ 1400-1320<br>▪ 3450-3225<br>▪ 1200-1105, 1280-1175, and 1375-1260<br>▪ 620-510, and 770-650	▪ Aliphatic ether or sulfonate salt, aliphatic alcohol, aliphatic sulfonylchloride, and organic halogen compounds
Acidified GB with 0.50 M acetic	▪ C-H stretch- alkyl<br>▪ C-H bend- CH_2/CH_3<br>▪ C-O stretch<br>▪ C-O bend<br>▪ O-H bend	▪ 2975-2845 and 3000-2855<br>▪ 1475-1415<br>▪ 1085-1000<br>▪ 500-400<br>▪ 1400-1300	▪ Aliphatic alcohol, aliphatic mercapto group and fluorine compounds

	▪ O-H stretch<br>▪ C-S stretch<br>▪ C-S, CH2 bend<br>▪ S-H stretch<br>▪ C-F stretch<br>▪ C-F bend	▪ 3450-3225<br>▪ 680-645<br>▪ 1290-1220<br>▪ 2565-2525<br>▪ 1200-1105, 1280-1175, and 1375-1260<br>▪ 620-510, and 770-650	
Acidified GB with 0.25 M phosphoric acid	▪ C-H stretch- alkyl<br>▪ C-H bend- CH_2<br>▪ C-H bend, CH_2/C-S<br>▪ C-O- stretch<br>▪ S-H stretch<br>▪ C=C and C=N stretch<br>▪ C=C and S=N stretch<br>▪ C-H bend<br>▪ C-S=O, sulfoxy stretch<br>▪ C-O bend<br>▪ C-O stretch<br>▪ SO_2 sulfoxy stretch<br>▪ O-H bend<br>▪ O-H stretch	▪ 3100-3010, 3000-2855, and 2870-2845<br>▪ 1470-1415<br>▪ 1450-1410 and 1305-1265<br>▪ 1120-1020<br>▪ 2565-2535<br>▪ 1520-1470, 1615-1570<br>▪ 1450-1410<br>▪ 1470-1415 and 1300-1200<br>▪ 1060-1020<br>▪ 500-400<br>▪ 1085-1000<br>▪ 1150-1115 and 1325-1275<br>▪ 1400-1300<br>▪ 3430-3225	▪ Pyridines, aliphatic ether or sulfoxide, aliphatic alcohol, aliphatic thiocompound, and hydroxy compound
Acidified GB with 0.50 M phosphoric acid	▪ C-H stretch<br>▪ N-H stretch<br>▪ N-H bend<br>▪ C-H bend, CH_2/CH_3<br>▪ C-N stretch<br>▪ SO_2 sulfoxy stretch<br>▪ C-O stretch<br>▪ C-O bend<br>▪ C-H bend<br>▪ C-F bend<br>▪ C-F stretch<br>▪ O-H bend<br>▪ O-H stretch	▪ 3015-2975, 2950-2910, and 2885-2845<br>▪ 3425-3270 and 3550-3345<br>▪ 1630-1590, 850-810, and 790-760<br>▪ 1475-1430<br>▪ 1130-1060<br>▪ 1205-1160 and 1065-1030<br>▪ 1210-1100<br>▪ 800-750<br>▪ 1480-1420 and 1390-1020<br>▪ 520-400<br>▪ 1090-1020<br>▪ 1400-1320<br>▪ 3450-3225	▪ Aliphatic amine, aliphatic ether or sulfonate salt, aliphatic alcohol, and organic halogen compounds
